# Update in Molecular Aspects and Diagnosis of Autoimmune Gastritis

**DOI:** 10.3390/cimb45070334

**Published:** 2023-06-21

**Authors:** Masaya Iwamuro, Takehiro Tanaka, Motoyuki Otsuka

**Affiliations:** 1 Department of Gastroenterology and Hepatology, Okayama University Graduate School of Medicine, Dentistry and Pharmaceutical Sciences, Okayama 700-8558, Japan; 2 Department of Pathology, Okayama University Graduate School of Medicine, Dentistry and Pharmaceutical Sciences, Okayama 700-8558, Japan

**Keywords:** autoimmune gastritis, esophagogastroduodenoscopy, genetic predisposition, lymphocyte, oxidative stress

## Abstract

Recent studies have advanced our understanding of the pathophysiology of autoimmune gastritis, particularly its molecular aspects. The most noteworthy recent advancement lies in the identification of several candidate genes implicated in the pathogenesis of pernicious anemia through genome-wide association studies. These genes include *PTPN22*, *PNPT1*, *HLA-DQB1*, and *IL2RA*. Recent studies have also directed attention towards other genes such as *ATP4A*, *ATP4B*, *AIRE*, *SLC26A7*, *SLC26A9*, and *BACH2* polymorphism. In-depth investigations have been conducted on lymphocytes and cytokines, including T helper 17 cells, interleukin (IL)-17A, IL-17E, IL-17F, IL-21, IL-19, tumor necrosis factor-α, IL-15, transforming growth factor-β1, IL-13, and diminished levels of IL-27. Animal studies have explored the involvement of roseolovirus and *H. pylori* in relation to the onset of the disease and the process of carcinogenesis, respectively. Recent studies have comprehensively examined the involvement of autoantibodies, serum pepsinogen, and esophagogastroduodenoscopy in the diagnosis of autoimmune gastritis. The current focus lies on individuals demonstrating atypical presentations of the disease, including those diagnosed in childhood, those yielding negative results for autoantibodies, and those lacking the typical endoscopic characteristics of mucosal atrophy. Here, we discuss the recent developments in this field, focusing on genetic predisposition, epigenetic modifications, lymphocytes, cytokines, oxidative stress, infectious agents, proteins, microRNAs, autoantibodies, serum pepsinogen, gastrin, esophagogastroduodenoscopy and microscopic findings, and the risk of gastric neoplasm.

## 1. Introduction

Autoimmune gastritis, also known as type A gastritis or autoimmune metaplastic atrophic gastritis, is a chronic inflammatory disorder of the stomach that arises from an autoimmune response directed toward parietal cells and intrinsic factor [1,2]. Parietal cells secrete hydrochloric acid (HCl), while intrinsic factor is essential for the absorption of vitamin B_12_. The pathophysiology of autoimmune gastritis involves the activation of autoreactive T cells that recognize and assault parietal cells and intrinsic factor. This immune response induces the destruction of parietal cells, culminating in decreased HCl secretion. This condition can lead to atrophic gastritis, intestinal metaplasia, and an increased risk of gastric cancer. Vitamin B_12_ is an essential nutrient that plays a crucial role in many physiological processes, including red blood cell formation, DNA synthesis, and nervous system functions. The impaired absorption of vitamin B_12_ is the primary cause of pernicious anemia, leading to various symptoms, including anemia and neurological problems, which can be severe if left untreated. Although numerous molecular mechanisms are involved in autoimmune gastritis, including genetic factors, autoantibodies, inflammatory cells, and pro-inflammatory cytokines, the complete pathophysiological mechanism remains to be fully elucidated.

There are several challenges in diagnosing autoimmune gastritis, including the absence of specific symptoms, the interpretation of biopsy specimens, the heterogeneity of the disease, and a paucity of authenticated biomarkers. The symptoms of autoimmune gastritis are nonspecific and overlap with other gastrointestinal disorders, such as peptic ulcer disease, functional dyspepsia, and acid reflux, impeding a diagnosis based on clinical presentation alone. Although a biopsy of the gastric mucosa is required to confirm autoimmune gastritis, obtaining adequate samples can be challenging, particularly in patients with mild or early-stage inflammation. However, the heterogeneity of autoimmune gastritis in terms of clinical and histological manifestations and severity poses difficulties in the development of screening methods and standardized diagnostic criteria.

Recent studies have advanced our understanding of the diagnosis and pathophysiology of autoimmune gastritis, particularly its molecular aspects. The primary aim of this review is to explore recent advancements in the pathophysiology and diagnosis of autoimmune gastritis.

## 2. Search Strategy

We conducted a systematic search of the PubMed database to retrieve all peer-reviewed articles published between 1 January 2020 and 6 February 2023, without imposing any study design filters [3]. To augment our search results, we manually screened for additional relevant articles using a reference list of selected publications that met our eligibility criteria. Our search strategy employed the keywords “autoimmune” and “gastritis”, and was executed by the principal investigator, Iwamuro M. Criteria for article inclusion were as follows: (1) peer-reviewed articles describing cases and animal and cell studies of autoimmune gastritis; and (2) review articles, original articles, case series, and case reports. Articles were excluded if they (1) did not focus primarily on autoimmune gastritis; (2) were letters, editorials, or correction notices; or (3) were written in languages other than English. All eligible articles were evaluated.

## 3. Search Results

**Figure 1:**
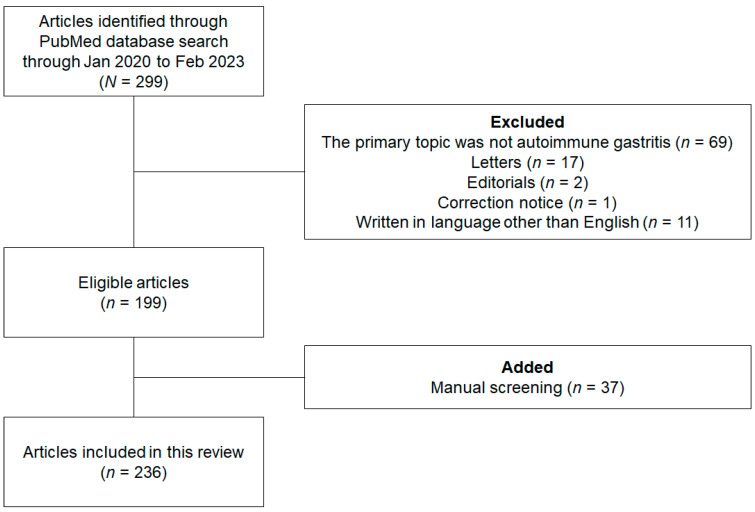
Flow diagram summarizing the identification, screening, eligibility checking, and exclusion processes of the literature search.

Figure 1 presents a flow diagram summarizing the identification, screening, eligibility, and exclusion processes of the literature search. The primary search yielded 299 studies. A subsequent screening revealed that autoimmune gastritis was not the primary focus of 69 of these articles. Articles that were letters (*n* = 17), editorials (*n* = 2), correction notices (*n* = 1), or not in English (*n* = 11) were then excluded. Ultimately, 199 articles were retrieved from the initial PubMed search after applying the exclusion criteria, including original articles (*n* = 98), case reports (*n* = 56), reviews (*n* = 44), and one conference poster. After a manual screening, 37 additional articles were deemed relevant and subsequently included in the review. A total of 236 articles were reviewed in detail.

## 4. Molecular Aspects of the Pathophysiology of Autoimmune Gastritis

### 4.1. Overview

**Figure 2:**
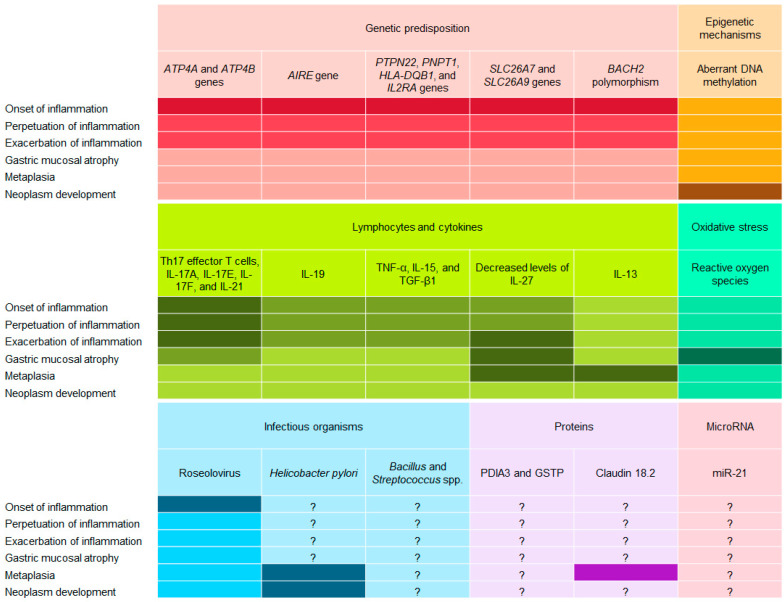
Potential pathophysiological mechanisms of autoimmune gastritis. The depicted associations of each factor are represented by varying degrees of color intensity. Question marks (?) signify that the extent of associations between each factor and the pathophysiological mechanisms of autoimmune gastritis remains ambiguous and/or undetermined. It should be noted that the authors of this article determined the colors of the grids, and the extent of these associations was not necessarily investigated in the original research studies.

Multiple molecular mechanisms have been implicated in the pathophysiology of autoimmune gastritis. The molecular mechanisms associated with the onset, perpetuation, and exacerbation of inflammation, gastric mucosal atrophy, metaplasia, and neoplasm development include genetic predispositions, epigenetic modifications, lymphocytes, cytokines, oxidative stress, infectious agents, proteins, and microRNAs. This section elucidates the potential processes and roles of these potential etiologies in autoimmune gastritis (Figure 2).

### 4.2. Genetic Predisposition and Epigenetic Mechanisms

Autoimmune gastritis has been shown to have a genetic predisposition, and several genes have been identified as risk factors for the disease. These genes include those involved in the maintenance of gastric acid secretion, such as ATPase H+/K+ transporting subunit alpha (*ATP4A*) [4] and beta (*ATP4B*) [5]. An *ATP4A* mutation knock-in mouse model exhibited gastric achlorhydria and the dysregulation of the acid–base equilibrium within gastric parietal cells, subsequently impairing mitochondrial biogenesis [6]. Dysfunctional mitochondria trigger reactive oxygen species (ROS) signaling, resulting in oxidative stress-induced damage, caspase-3-mediated apoptosis, inflammation, and atrophy of parietal cells.

The causative genes of autoimmune gastritis have been primarily identified in individuals diagnosed with autoimmune polyendocrine syndrome (APS). APS is a relatively uncommon autoimmune condition characterized by the development of multiple endocrine organ-specific autoimmune diseases, with autoimmune gastritis being one of the most prevalent manifestations. The autoimmune regulator (*AIRE*) plays a critical role in the development of APS type 1 [7]. The *AIRE* gene encodes a transcription factor expressed in the thymus that plays a key role in the negative selection of autoreactive T cells. Mutations in the *AIRE* gene lead to a failure to eliminate self-reactive T cells. A survey conducted in Italy on 158 patients with APS type 1 demonstrated that various *AIRE* mutations were present in a significant proportion of the tested alleles. Specifically, R139X was observed in 21.3%, R257X in 11.8%, W78R in 11.4%, C322fsX372 in 8.8%, T16M in 6.2%, R203X in 4%, and A21V in 2.9% of the patients [8].

Genome-wide association studies conducted on 2166 cases of pernicious anemia and 659,516 European controls from population-based biobanks identified genes associated with pernicious anemia [9]. These genes are responsible for immune regulation and include protein tyrosine phosphatase non-receptor type 22 (*PTPN22*), polyribonucleotide nucleotidyltransferase 1 (*PNPT1*), major histocompatibility complex class II, DQ beta 1 (*HLA-DQB1*), interleukin 2 receptor subunit alpha (*IL2RA*), and the *AIRE* genes. A study using whole exome sequencing to investigate five families with autoimmune thyrogastric disorders identified distinct pathogenic variants in solute carrier family 4 member 2 (*SLC4A2*), solute carrier family 26 member 7 (*SLC26A7*), and member 9 (*SLC26A9*) genes [10]. These solute carriers play a pivotal role in modulating the acid–base balance within parietal cells, thus serving as essential contributors to the H+/K+ exchange.

Recent studies demonstrated the potential involvement of BTB and CNC homology 2 (*BACH2*) polymorphisms in the pathogenesis of autoimmune gastritis [11]. Additionally, the aberrant methylation of promoter CpG islands in tumor suppressor genes related to the cell cycle, cell adhesion, p53, and Wnt pathways has been observed in patients with autoimmune gastritis [12].

### 4.3. Lymphocytes and Cytokines

CD4^+^ and CD8^+^ T cells, along with macrophages and B cells, participate in the inflammatory process underlying autoimmune gastritis. Among these, T helper 1 (Th1) cells secrete the pro-inflammatory cytokine interferon-gamma (IFN-γ), which contributes to the destruction of parietal cells and plays a critical role in the pathogenesis of autoimmune gastritis [13]. The primary autoantigen in autoimmune gastritis is H+/K+-ATPase, whose recognition by gastric T cells stimulates the secretion of Th1 cytokines. Consequently, the cytotoxic destruction of parietal cells is mediated by perforin and Fas ligands, culminating in gastric atrophy [14]. Recent studies have identified a predominant response of Th17 effector T cells in autoimmune gastritis. Th17 cells secrete interleukin (IL)-17 and IL-21 and play a role in host defense against extracellular pathogens, particularly at the mucosal and epithelial barriers; however, the aberrant activation of these signals has been linked to the pathogenesis of various autoimmune diseases. Th17 cells are present in the gastric mucosa [15,16,17]. Researchers have also revealed that H+/K+-ATPase can activate lymphocytes in the gastric lamina propria, which line the mucous membranes within the stomach. This activation culminates in the expansion of the lymphocyte population and the induction of elevated levels of IL-17A and IL-17F [18]. A significant elevation in the serum concentrations of IL-17A, IL-17E, IL-17F, and IL-21 was also evident in patients with autoimmune gastritis compared with healthy controls. These results confirm the relevance of Th17 cells in the pathogenesis of autoimmune gastritis.

A study on the cytokine profile of patients with pernicious anemia demonstrated a notable elevation in their serum IL-19 levels compared with healthy controls [19]. Furthermore, an in vitro investigation of gastric lamina propria mononuclear cells derived from patients with pernicious anemia revealed their capacity to produce augmented levels of IL-19. Another study found that the number of plasma cells significantly increased in a subset of lamina propria mononuclear cells [20]. In vitro culture using biopsy specimens from patients with autoimmune gastritis revealed higher concentrations of tumor necrosis factor-alpha (TNF-α), IL-15, and transforming growth factor-beta 1 (TGF-β1) compared with those from healthy controls [20].

The TxA23 mouse strain is widely used as an animal model to investigate the pathophysiology of autoimmune gastritis [21]. This mouse strain harbors transgenic T-cell receptors in their CD4^+^ T cells that recognize a specific peptide derived from the H+/K+ ATPase α chain, which is selectively expressed by parietal cells. Recent research revealed the presence of IL-27-secreting macrophages and dendritic cells in the corpus of TxA23 mice [22]. Mice deficient in IL-27 display more pronounced gastritis, atrophy, and spasmolytic polypeptide-expressing metaplasia (SPEM) than their wild-type counterparts. Conversely, the administration of recombinant IL-27 considerably diminished the severity of inflammation, atrophy, and SPEM. These observations imply that IL-27 serves as an inhibitor of gastritis and SPEM within the gastric mucosa. Another investigation using TxA23 mice revealed that IL-13, which is predominantly produced by mast cells within the inflamed gastric mucosa, directly induces the progression of metaplastic changes, such as SPEM, in epithelial cells [23].

The discrepancies observed in the outcomes across studies may stem from differences in the methodologies or divergences among patient cohorts. Nonetheless, previous research suggests that various inflammatory cells and cytokines may intricately and interactively participate in the inception of inflammation, parietal cell damage, and metaplasia in the gastric mucosa of patients with autoimmune gastritis.

### 4.4. Oxidative Stress

Oxidative stress is the state of disequilibrium between the generation of ROS and the ability of an organism to detoxify them. ROS can directly damage the gastric mucosa, leading to parietal cell destruction and atrophic gastritis. An investigation using TxA23 mice revealed that the acid–base equilibrium within parietal cells impacts the process of mitochondrial biogenesis. Thus, an acid–base imbalance leads to the malfunctioning of mitochondria and the stimulation of ROS signaling, consequently initiating caspase-3-mediated apoptosis in parietal cells [6].

The thiol-disulfide ratio is used to evaluate oxidative stress in the body by measuring the balance between the reduced (thiol) and oxidized (disulfide) forms of thiol-containing molecules. A reduced thiol-to-disulfide ratio indicates a higher degree of oxidative stress. The thiol-disulfide ratio was found to be reduced in patients with autoimmune gastritis compared with healthy controls [24]. These observations highlighted the role of oxidative stress as a contributing component in the pathogenesis of autoimmune gastritis.

### 4.5. Infectious Organisms

Although a genetic predisposition appears to play a critical role in the development of autoimmune gastritis, environmental factors may also play a role. Several infectious organisms, including viruses and bacteria, are implicated in the pathogenesis of autoimmune gastritis.

A study in BALB/c mice demonstrated that neonatal infection with murine roseolovirus could elicit autoimmune gastritis in adult mice in the absence of ongoing infection [25]. This roseolovirus-induced gastritis was found to rely on replication during the neonatal period as well as CD4^+^ T cells and IL-17. Moreover, early-life roseolovirus infection disrupts central tolerance and triggers autoimmune gastritis. Therefore, human roseoloviruses, such as herpesviruses 6A, 6B, and 7 (HHV-6A, HHV-6B, and HHV-7, respectively), have the potential to initiate autoimmune gastritis.

*Helicobacter pylori* is the main cause of chronic atrophic gastritis and is considered a potential trigger for gastric autoimmunity, leading to the development of autoimmune gastritis [26]. However, some studies suggest that *H. pylori* infection is inversely related to autoimmune gastritis, as it induces chronic inflammation and atrophic changes in the stomach, which may prevent the development of autoimmune gastritis [27]. Therefore, the relationship between *H. pylori* and autoimmune gastritis remains complex and is currently being studied.

Individuals diagnosed with pernicious anemia have a seven-fold higher relative risk of developing gastric cancer, with a yearly incidence rate of 0.27% per person [28,29,30]. Nonetheless, a recent prospective observational study of 211 *H. pylori*-negative patients with autoimmune gastritis reported no incidence of gastric cancer during a mean follow-up of 7.5 years [31]. The increased risk of gastric cancer observed in patients with autoimmune gastritis could be attributed to unrecognized previous or current *H. pylori* infections.

Analyses of gastric bacterial taxa revealed a statistically significant surge in the prevalence of *Bacillus* and *Streptococcus* spp. in patients with autoimmune gastritis compared with those without [32]. In particular, *Bacillus cereus* is an exclusive constituent of the gastric microbiota in patients with autoimmune gastritis-related gastric cancer patients [33]. Mechanisms such as direct and bacterial metabolite-induced tumorigenesis, alterations in the stomach microenvironment, and disruptions in tumor immunity may underlie the initiation of stomach-specific carcinogenesis.

### 4.6. Proteins

Two-dimensional difference gel electrophoresis, combined with immunoblotting, confirmed that the levels of gene products, including ATP synthase subunit alpha (*ATP5F1A*), pepsinogen A3 (*PGA3*), succinate dehydrogenase complex iron sulfur subunit B (*SDHB*), and progastricsin (*PGC*), were found to be decreased in patients with autoimmune gastritis [34]. Conversely, the abundance of proteins associated with the gene products of protein disulfide isomerase family A member 3 (*PDIA3*) and glutathione S-transferase pi 1 (*GSTP*) is significantly increased in the gastric mucosa of these patients. PDIA3 functions as a redox sensor by stimulating the mammalian mechanistic target of rapamycin complex 1 (mTORC1), which in turn facilitates the assembly of mTOR complexes to enable cellular adaptation to oxidative stress. Hence, increased levels of PDIA3 protein may indicate oxidative stress in autoimmune gastritis. GSTP proteins are presumed to participate in xenobiotic metabolism and modulate susceptibility to cancer.

Claudin 18.2 has a higher expression in intestinal-type gastric cancers than in other cancer subtypes [35]. Immunohistochemical analyses targeting claudin 18 revealed that 18.9% of autoimmune gastritis cases with intestinal metaplasia manifest positive staining, in contrast to 71.4% in those without autoimmune gastritis, indicating the distinct nature of intestinal metaplasia in autoimmune gastritis [36].

### 4.7. microRNA

MicroRNA (miR)-21 has been extensively studied in relation to gastric cancer and is one of the most commonly upregulated microRNAs in this malignancy [37]. miR-21 targets and inhibits the expression of several tumor suppressor genes. Thus, miR-21 plays a crucial role in promoting tumor growth, invasion, and metastasis by regulating various biological processes. The levels of circulating miR-21 were found to be elevated in patients with autoimmune gastritis and *H. pylori*-associated atrophic gastritis compared with those in healthy controls, whereas miR223 levels were reduced in these individuals [38]. These findings suggest that miR-21 is involved in the pathogenesis of both autoimmune gastritis and *H. pylori*-associated atrophic gastritis, potentially serving as a shared mechanism between these two conditions.

## 5. Diagnosis of Autoimmune Gastritis

### 5.1. Overview

Currently, internationally standardized diagnostic criteria for autoimmune gastritis are lacking. Diagnosing autoimmune gastritis traditionally relies on clinical and laboratory findings, necessitating confirmation by identifying characteristic histopathological alterations [28,39]. The laboratory data used for screening and/or diagnosis included the presence of autoantibodies and the serum levels of pepsinogen and gastrin.

### 5.2. Autoantibodies

The hallmark feature of autoimmune gastritis is the presence of antibodies against parietal cells and intrinsic factor, with the latter being deemed more discriminative in diagnostic specificity [40]. The presence of autoantibodies against H+/K+-ATPase is thought to play a critical role in the pathogenesis of autoimmune gastritis. A previous study demonstrated that the evaluation of intrinsic factor antibody levels using an enzyme-linked immunosorbent assay exhibited a sensitivity of 27%, coupled with an absolute specificity of 100%. Conversely, parietal cell antibodies displayed a considerable sensitivity of 81% and a specificity of 90%, effectively distinguishing patients with autoimmune gastritis [41,42]. However, it should be noted that a gradual decline in the titer of parietal cell antibodies occurs from the early phase to the end phase of autoimmune gastritis [43]. In general, the presence of anti-parietal cell antibodies serves as an indication of the initial phases of autoimmune gastritis, whereas the detection of anti-intrinsic factor antibodies becomes more pertinent during the advanced stages, characterized by a significant depletion of parietal cells and a subsequent deficiency in intrinsic factor.

Researchers have developed luciferase immunoprecipitation assays targeting ATP4A and ATP4B and have demonstrated favorable diagnostic accuracy in patients with histologically confirmed autoimmune gastritis [44]. The sensitivities of the ATP4A and ATP4B assays were 75% and 77%, respectively, with corresponding specificities of 88% for both tests [45]. Notably, partial ROC-pAUC90 analysis indicated a superior diagnostic performance for the ATP4B assay. In contrast, luciferase immunoprecipitation assays directed against intrinsic factor antibodies exhibited a diagnostic sensitivity of 32% and specificity of 95% in identifying autoimmune gastritis [46].

### 5.3. Serum Pepsinogen

In autoimmune gastritis, the pathophysiological process encompasses progressive oxyntic gastric atrophy, which entails the destruction of gastric chief cells, ultimately resulting in the diminished synthesis of hydrochloric acid and pepsinogens. As a characteristic feature, patients with autoimmune gastritis commonly exhibit a reduction in their pepsinogen I (PG I)/pepsinogen II (PG II) ratio, primarily attributed to the reduced secretion of PG I. A study demonstrated the superiority of the PG I/PG II ratio (<2.1) over individual PG I or PG II measurements in diagnosing autoimmune gastritis in both its florid and end stages, exhibiting a remarkable sensitivity of 1.00 and a specificity of 0.95 [47]. Another study demonstrated that a PG I/PG II ratio below 1.8 exhibited a sensitivity of 96.3% and a specificity of 67.7% [48]. A study conducted in Turkey revealed that a PG I/PG II ratio ≤ 1.9 demonstrated a sensitivity of 100% and a specificity of 100% in the diagnosis of autoimmune gastritis [49]. Therefore, the PG I/PG II ratio may be a viable serological screening tool for autoimmune gastritis. Recent research has revealed that the levels of PG I and the ratio of PG I to PG II were substantially diminished in autoimmune gastritis patients exhibiting extensive atrophy, encompassing a mixed form of autoimmune and *H. pylori*-associated gastritis, when juxtaposed with those displaying corpus-restricted atrophy [50]. These findings suggest that the PG I level and the PG I/PG II ratio exhibit variations based on the existence of *H. pylori* infection, even within the same cohort of autoimmune gastritis patients.

GastroPanel^®^, a commercially available serological biomarker panel, is widely used in numerous countries, with a particularly significant prevalence in Europe [51,52,53]. The panel comprised PG I, PG II, gastrin-17, and *H. pylori* antibodies. Employing this panel facilitates screening for *H. pylori*-associated gastritis and autoimmune gastritis, where decreased serum PG I levels and/or low PG I/PG II ratios indicate autoimmune gastritis.

### 5.4. Gastrin

In autoimmune gastritis, reduction in gastric acid secretion triggers a compensatory response in the body, resulting in an increase in gastrin levels, a hormone that stimulates the release of gastric acid from parietal cells. A gradual decline in serum gastrin levels was observed, exhibiting a stepwise decrease from the early phase to the advanced stages of the disease [42]. Measuring gastrin levels can aid in the diagnosis and monitoring of autoimmune gastritis. A preceding study has established the diagnostic significance of gastrin levels surpassing 355 pg/mL in the identification of autoimmune gastritis [47]. Nevertheless, an alternative study proposes that the most effective combination of noninvasive biomarkers for detecting corpus-restricted atrophic gastritis consists of PGI/II with anti-parietal cell antibodies, whereas the inclusion of gastrin levels offers minimal utility in clinical applications [54].

Gastrin is considered to play a pivotal role in the development and progression of gastric neuroendocrine tumors. The principal mechanism underlying gastrin’s involvement in neuroendocrine tumor development is its stimulatory effect on the growth of enterochromaffin-like cells within the stomach. In a previous study, it was found that employing a pepsinogen I/II ratio below 2.3 and gastrin-17 levels exceeding 29.6 pmol/L serves as a definitive approach to distinguish autoimmune gastritis patients with gastric neuroendocrine tumors from those without [55]. By synergistically applying these established thresholds, an impressive accuracy rate of 88.9% (16 out of 18) was achieved in accurately identifying autoimmune gastritis patients with gastric neuroendocrine tumors.

### 5.5. Esophagogastroduodenoscopy

Observations made through esophagogastroduodenoscopy and histological assessments of endoscopic biopsy specimens play pivotal roles in the diagnosis of autoimmune gastritis. Corpus-predominant atrophy, defined as a state distinguished by substantial or predominant atrophy within the gastric corpus while sparing the gastric antrum, is the principal hallmark of autoimmune gastritis. Studies have revealed distinctive endoscopic features associated with this disease. An endoscopic assessment of 245 Japanese patients diagnosed with autoimmune gastritis revealed various findings, including sticky adherent dense mucus (32.4%), scattered minute whitish protrusions (32.0%), remnants of oxyntic mucosa (31.5%), patchy redness (22.1%), circular wrinkle-like patterns (22.1%), hyperplastic polyps (21.1%), and red streaks (10.4%) [56]. In recent years, certain reports have directed their attention toward early-stage autoimmune gastritis as opposed to conventional autoimmune gastritis, which is characterized by prominent mucosal atrophy in the gastric body. Possible prevalent endoscopic manifestations associated with early-stage autoimmune gastritis have been documented, including a mosaic pattern with a slight swelling of the gastric area, diffuse reddened and edematous gastric fundic gland mucosa, and pseudopolyp-like nodules [57].

Our research team focused on analyzing the lymphocyte composition within the gastric mucosa of patients with autoimmune gastritis [58]. Gastric mucosal lymphocytes were isolated from both the gastric body and antrum regions, and an investigation was carried out to assess the proportions of CD8^+^ and CD4^+^ cells within the T-cell population (CD3^+^ cells). The division of the CD4^+^/CD3^+^ ratio in the gastric body by the CD4^+^/CD3^+^ ratio in the gastric antrum (body CD4^+^/antrum CD4^+^) revealed a significantly higher value in cases of autoimmune gastritis compared with *H. pylori*-associated gastritis. Similarly, dividing the CD8^+^/CD3^+^ ratio by the CD4^+^/CD3^+^ ratio in the gastric antrum (antrum CD8^+^/CD4^+^) yielded a higher value for autoimmune gastritis than for *H. pylori*-associated gastritis. Receiver-operating characteristic analysis demonstrated the superior effectiveness of antrum CD8^+^/CD4^+^ in distinguishing autoimmune gastritis from *H. pylori*-associated gastritis, as compared with body CD4^+^/antrum CD4^+^. An antrum CD8^+^/CD4^+^ ratio exceeding 4.0 exhibited a sensitivity of 71.4% and a specificity of 93.3% in diagnosing autoimmune gastritis. Given the subjective nature of endoscopic findings, our research suggests that lymphocyte composition may serve as an objective and potential diagnostic marker for autoimmune gastritis.

### 5.6. Microscopic Findings

Autoimmune gastritis is characterized by specific histopathological alterations. Typical features include the chronic inflammation of the gastric mucosa, lymphocytic infiltration, the destruction of parietal cells, and glandular atrophy. These distinct histological changes are indicative of the autoimmune nature of the disease and aid in its diagnosis and classification. Histopathologically, autoimmune gastritis has been classified into early, florid, and end stages [2,59,60]. These stages represent distinct phases of disease progression, each characterized by specific histological features. In the early stage, there is evidence of chronic inflammation and lymphocytic infiltration within the gastric mucosa. Parietal cell destruction and glandular atrophy may be minimal or absent at this stage. The florid stage is marked by more pronounced inflammation, extensive lymphocytic infiltration, and the significant destruction of parietal cells. Glandular atrophy becomes more prominent, and there may be the presence of metaplastic changes, such as SPEM. In the end stage of autoimmune gastritis, there is severe inflammation, extensive glandular atrophy, and a marked loss of parietal cells. Metaplastic changes are often widespread, including the development of metaplasia and dysplasia. These histopathological differences between the stages of autoimmune gastritis reflect the progressive nature of the disease and provide insights into its pathogenesis and clinical implications.

### 5.7. Risk of Gastric Neoplasm

Among individuals diagnosed with autoimmune gastritis, two primary categories of gastric neoplasms can potentially arise, namely gastric adenocarcinoma and neuroendocrine tumors. The identification of neoplastic lesions through esophagogastroduodenoscopy is crucial for monitoring patients with autoimmune gastritis. Among a cohort of 135 Chinese patients diagnosed with autoimmune gastritis, the following clinical manifestations were observed: multiple type 1 gastric neuroendocrine tumors (37.0%), gastric hyperplastic polyps exhibiting neoplastic transformation (11.1%), high-grade dysplasia or adenocarcinoma (5.9%), and low-grade dysplasia or adenoma (3.7%) [61]. In a Japanese cohort, the prevalence was 11.4% for type 1 neuroendocrine tumors and 9.8% for adenocarcinomas [56]. A distinct pattern was also observed in the distribution of tumors in patients with gastric cancer associated with autoimmune gastritis [62]. Regarding the spatial sites of gastric cancer, 28.1%, 53.1%, and 18.8% were located in the upper, middle, and lower regions, respectively. Morphologically, these tumors were categorized as flat depressed (46.9%), flat elevated (28.1%), flat (15.6%), or elevated (9.4%). Gastric cancers in patients with autoimmune gastritis exhibit a notable prevalence of protruding tumor types, larger tumor sizes, and histopathological papillary tumors specifically localized in the upper region of the stomach.

It is advisable for patients with autoimmune gastritis to undergo endoscopic examination within 3–5 years, as this serves as a means to identify neoplastic lesions [30,63]. In an Italian study involving 160 patients who underwent a comprehensive 3-year endoscopic follow-up comprising 122 individuals with autoimmune gastritis and 38 with multifocal atrophic gastritis, notable findings emerged [63]. The investigation identified three gastric cancers, four instances of low-grade dysplasia, two low-grade dysplasia adenomas, and seven type 1 neuroendocrine tumors. It is worth noting that all detected lesions were effectively managed through either endoscopic or surgical intervention, resulting in favorable outcomes. These findings provide compelling evidence to support the appropriateness of the 3-year endoscopic follow-up protocol.

## 6. Autoimmune Gastritis without Typical Features

While autoimmune gastritis is commonly diagnosed in adult patients exhibiting seropositivity for autoantibodies, recent studies have also directed attention toward individuals who lack typical characteristics of the disease, including those diagnosed during childhood and those who test negative for autoantibodies.

An observational study conducted in Italy on a cohort of 51 patients presenting with potential autoimmune gastritis, characterized by the presence of serum anti-parietal cell antibody positivity and the absence of gastric histopathological alterations, revealed that 47.1% of these subjects transitioned to overt autoimmune gastritis within a median time frame of 2 years [64]. The presence of a concurrent autoimmune disorder emerged as a substantial risk factor for progression to overt autoimmune gastritis (hazard ratio: 4.09). Another study conducted by the same research team revealed that an increased number of CD3^+^ lymphocytes within the deep regions of the oxyntic glands might serve as an indicative marker for potential autoimmune gastritis [65].

A recent investigation revealed that the prevalence of autoimmune gastritis among pediatric patients who underwent gastric biopsies was determined to be 0.15% [66]. These pediatric patients were diagnosed at an average age of 10.9 years and were predominantly female (68.2%). Most patients (59.1%) exhibited extragastric immune disorders. The long-term prognosis in children with autoimmune gastritis remains unexplored [67].

## 7. Conclusions

Autoimmune gastritis is a complex autoimmune disease characterized by the destruction of gastric parietal cells and the development of atrophic gastritis. Recent investigations have established a correlation between genetic predisposition, which augments susceptibility to this condition, and the potential implications of epigenetic modifications, lymphocytes, cytokines, oxidative stress, infectious agents, proteins, and microRNAs in its pathophysiological processes. Subsequent studies are imperative to unravel the mechanisms underlying the onset, perpetuation, and progression of inflammation and neoplasia in patients with autoimmune gastritis.

## Data Availability

Data sharing is not applicable to this article as no new data was created or analyzed in this study.
